# Venom-Derived Neurotoxins Targeting Nicotinic Acetylcholine Receptors

**DOI:** 10.3390/molecules26113373

**Published:** 2021-06-03

**Authors:** Ayaulym Bekbossynova, Albina Zharylgap, Olena Filchakova

**Affiliations:** Biology Department, School of Sciences and Humanities, Nazarbayev University, Kabanbay Batyr Ave., 53, Nur-Sultan 010000, Kazakhstan; ayaulym.bekbossynova@nu.edu.kz (A.B.); albina.zharylgap@nu.edu.kz (A.Z.)

**Keywords:** nAChR, α-conotoxins, three-finger α-neurotoxins

## Abstract

Acetylcholine was the first neurotransmitter described. The receptors targeted by acetylcholine are found within organisms spanning different phyla and position themselves as very attractive targets for predation, as well as for defense. Venoms of snakes within the *Elapidae* family, as well as those of marine snails within the *Conus* genus, are particularly rich in proteins and peptides that target nicotinic acetylcholine receptors (nAChRs). Such compounds are invaluable tools for research seeking to understand the structure and function of the cholinergic system. Proteins and peptides of venomous origin targeting nAChR demonstrate high affinity and good selectivity. This review aims at providing an overview of the toxins targeting nAChRs found within venoms of different animals, as well as their activities and the structural determinants important for receptor binding.

## 1. Introduction

### 1.1. Structural Features of Nicotinic AChRs for Ligand Interaction

Nicotinic acetylcholine receptors (nAChRs) are channel-coupled membrane receptors activated endogenously by acetylcholine (ACh) [1]. Together with ionotropic GABA (gamma-aminobutiric acid), glycine, 5-HT3 (5-hydroxytryptamine), and zinc activated ion channels, they belong to a cys-loop superfamily of ligand-gated ion channels [2]. The receptors are characterized by radial symmetry and have pentameric organization, with five subunits arranged radially around a central ion-conducting pore. The ACh binds at the interface between adjacent subunits. There are 17 subunits described in vertebrates: α1 to α10, β1 to β4, γ, δ, and ε. The α8 subunit is found in the avian genome [3] and is not present in the human genome. The difference between α and β subunits lies within the structure of the ligand-binding site—an α-like subunit is considered to contribute to the ligand-binding site with a principal (+) subunit interface, which includes loops A, B, and C, whereas a β-like subunit is considered a complementary (−), contributing with loops D, E, and F to the ligand-binding interface. The α2, α3, α4, and α6 subunits, through their association with β2 or β4 subunits, can form heteromeric receptors, with different stoichiometries of 2(α)3(β) or 3(α)2(β). More than one type of α and/or β subunits within a receptor could increase receptor diversity. α5 and β3 subunits are considered auxiliary subunits, not contributing to the ACh binding site but influencing properties of the receptor [4,5]. α7 and α9 subunits can assemble into a homomeric receptor. However, association with other subunits is also possible. For example, α7 can associate with β2 [6], while α9 associates with the α10 subunit [7]. α1, β1, δ, ε, and γ are subunits within muscle-type of nAChR, with ε subunits present in adult receptor type, while γ subunit present in fetal receptor.

**Figure 1:**
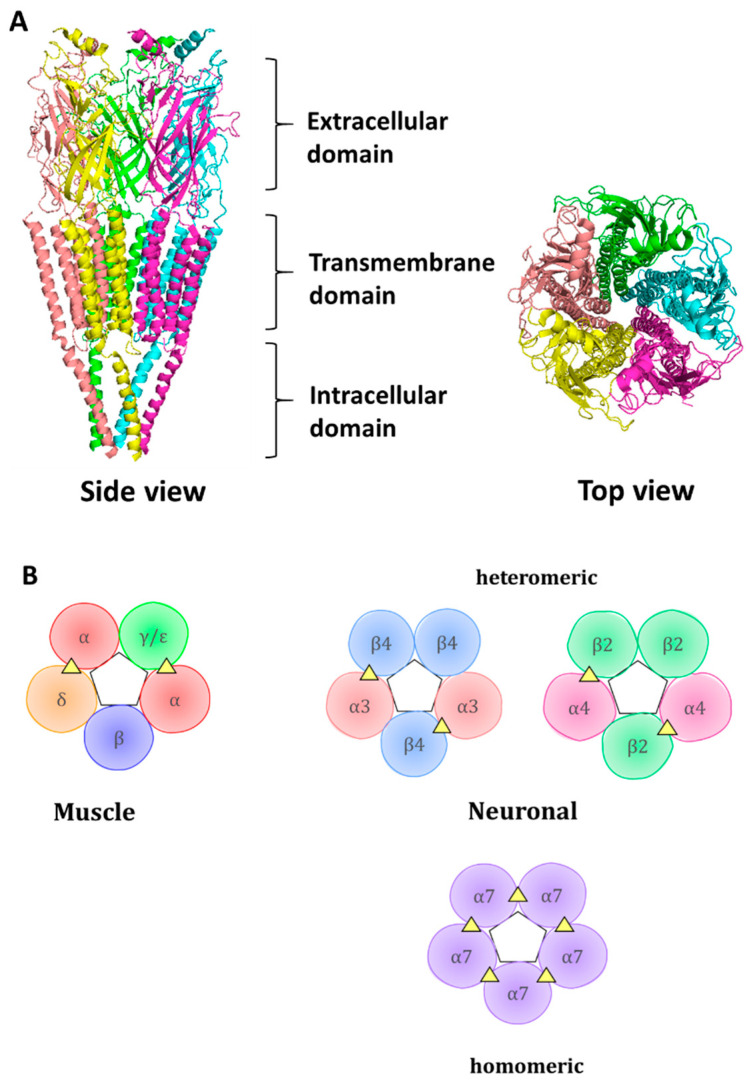
Representation of the structure of nAChR: (**A**) Cartoon structure of a muscle-like nAChR with pentameric organization shown through side and top views. Ligand-binding extracellular domain, pore-forming transmembrane domain, and an intracellular domain are shown. PDB accession number is 2BG9. (**B**) Cartoon representation of assembled muscle and neuronal nAChR subtypes. The pentameric organization of the receptor is shown. The triangles represent the subunit interfaces where endogenous ligands and competitive antagonists bind. In the muscle receptor, a γ-subunit is present in fetal form, while an adult receptor contains the ε- subunit. Two ligand-binding sites include α/δ and α/ε or α/γ interfaces, where the α subunit is considered principal, contributing with loops A, B, and C, whereas δ, ε, and γ are complimentary subunits, contributing with loops D, E, and F to the ligand-binding interface.

Each subunit contains an N-terminal extracellular domain (ECD) which contributes to the formation of an orthosteric ligand-binding site, a transmembrane domain containing four α-helices, out of which a second helix contributes to channel pore formation, and an intracellular domain (Figure 1). The orthosteric ligand-binding site is formed at the junction between two neighboring subunits, where loops A, B, and C, from principal subunits and loops D, E, and F, coalesce and form a hydrophobic cage from aromatic residues that provides a space for ACh binding. The aromatic residues protruding to the ligand-binding site are rather conserved; within the muscle α1 subunit, they include Tyr 93 from loop A, Trp 149 from loop B, and Tyr 190 and Tyr 198 from loop C. These aromatic residues within the principal subunit are assisted by aromatic residues of a complementary subunit, such as Trp 57 of the γ subunit [8]. The conserved aromatic residues interact with ligands via cation-π interaction.

The receptor structure was studied at an atomic level. The quest for receptor structure determination was facilitated through studies on acetylcholine-binding protein (AChBP), a soluble multimeric protein secreted by snail glial cells which buffers ACh concentration [9].

### 1.2. Importance of Nicotinic Acetylcholine Receptors in Physiology and Pathology

Acetylcholine is a widely distributed excitatory neurotransmitter. Within the human body, it is present in both branches of the autonomic nervous system: within the parasympathetic system in pre- and postganglionic cells, and within the sympathetic system in preganglionic cells. It is also a neurotransmitter at the periphery within the neuromuscular junction. nAChRs are widely distributed within the central nervous system (CNS) and peripheral nervous system, as well as outside the nervous system. The nucleus basalis of Meynert consists of cholinergic neurons widely projecting into different brain locations [10]. The most prevalent receptor subtype within the brain is the α4β2-containing receptor, the upregulation of which is observed following chronic nicotine consumption [11]. Existing in two stoichiometric forms ((α4)_2_(β2)_3_ and (α4)_3_(β2)_2_), α4β2-containing receptors can vary in their sensitivities to ACh [12]. nAChRs play an important role in the regulation of neurotransmitter release within central synapses, in activating the muscle at periphery. They play critical role within reward pathways, in particular, within nigrostriatal dopaminergic circuitry [13]. Recent discoveries have expanded the potential role of the receptors, and their significance was shown for pain signal transmission, particularly for α9α10 nAChR [14,15,16]. The receptors play a key role in an anti-inflammatory pathway, with an involvement of immune cells and vagal nerve activation [17].

Disfunction in nAChRs within neuromuscular junctions can lead to myasthenic syndrome. Within the CNS, abnormal functioning of the receptors can manifest itself as Alzheimer’s disease, schizophrenia, depression, and Parkinson’s disease [18]. Outside the CNS, improper functionality of the receptors can lead to skin disorders such as pemphigus vulgaris and cancer.

Thus, knowledge about the functionality of the receptors is of paramount importance. Ligands targeting the receptors can help decipher physiological mechanisms, as well as prevent pathological processes where receptors are involved [19,20]. Natural products and, in particular, venoms of venomous animals, such as snails, snakes, spiders, and scorpions, contain biologically active compounds targeting nAChRs; thus, they are actively and thoroughly investigated [21].

## 2. Snail Venoms as a Source of Toxins Acting on Acetylcholine Receptors

Predatory snails within the *Conus* genus produce peptides—conotoxins that target different ion channels [22]. Conotoxins differ in their number of disulfide bonds. The peptides with a general structure CC-Xm-C-Xn-C, where C represent cysteines, Xm and Xn—variable number of residues between cysteines—constitute the family of α-conotoxins (α-Ctx). α-Conotoxins are competitive antagonists of nAChRs [23,24]. The different number of residues within two loops contributes to the division of the α-conotoxins into several subgroups (3/5, 4/3, 4/4, 4/5, 4/6, and 4/7). Structurally, α-conotoxins have very rigid three-dimensional structure due to the restraints imposed by disulfide bonds. Many α-conotoxins have a conserved proline in loop 1 between Cys 1–Cys 3, which contributes to structural stability. α-conotoxins with four residues in loop 1 form a small 3_10_ α-helical region. Functionally, α-conotoxins could be divided into two groups: those targeting muscle-type nAChRs (α3/5 conotoxins) and those targeting neuronal nAChRs (α4/3, α4/4, α4/5, α4/6 and α4/7 conotoxins).

### 2.1. α3/5 Conotoxins

α3/5 conotoxins predominantly target muscle subtypes of nAChRs [25]. The two most well-studied of them include MI and GI conotoxins. α-Conotoxins MI, GI, and SIA have been shown to exhibit >10,000-fold selectivity for α/δ over the α/γ site in mammalian receptors (Table 1). However, the reverse pattern is observed in *Torpedo* receptors. Conserved Pro 6 and Tyr 12 residues are critical for hydrophobic interaction between the δ subunit and MI [26].

**Figure 2:**
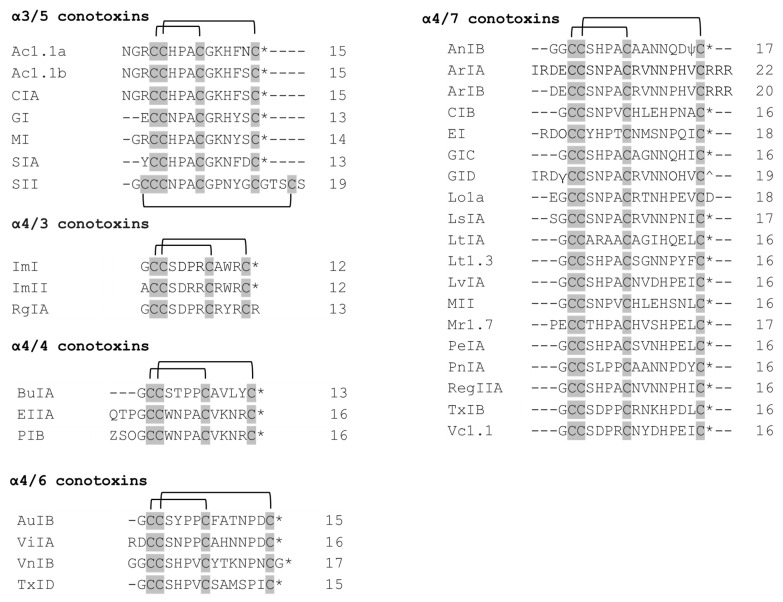
Sequence alignment of the α-conotoxins listed in a Table 1. The α-conotoxins with 3/5, 4/4, 4/6, and 4/7 spacing. The cysteines residues are shaded in gray and intrachain disulfide bonds are shown. The number of residues in each toxin is given to the right. * C—terminal carboxamide, ^ C—terminal carboxylate, ψ—sulfated tyrosine, *O*—hydroxyproline, *Z*—pyroglutamate.

Ac1.1a and Ac1.1b are almost identical α3/5 conotoxins differing in only one residue at position 14 [27] (Figure 2). They exhibit selectivity towards α/δ over α/γ site. The first three residues before the Cys 4 have been shown to lack an effect on binding the target.

α-Conotoxin GI blocks muscle-type nAChRs, with selectivity for the α/δ site in mouse muscle receptors [28], and the α/γ site in *Torpedo californica* electric organ receptors [29]. Pro 5, Gly 8, Arg 9, and Tyr 11 are critical residues for GI to target mouse α1β1δε nAChRs. [E1A] GI mutant demonstrated a three-fold increase in potency in mouse α1β1δε and a decreased potency in rat neuronal α9α10 nAChR, compared to wildtype GI [30].

NMR structures for both CIA and CIB alpha-conotoxins derived from *Conus catus* have been studied. α-Conotoxin CIA caused muscle paralysis in fish when tested in vivo [31], while 4/7 α-Conotoxin CIB blocked neuronal type nAChRs.

Overall, there are two conserved regions (residues 2 and 3 in the first cysteine loop, as well as residues 1, 2, and 4 in the second cysteine loop) inherent to α3/5 conotoxins (Figure 2), which include important residues for the binding to muscle-type nAChR. Notably, SII differs from the rest in two main ways: (1) it has Pro residue in place of expected Arg/Lys; (2) it has a long C-terminus. Structure–functional studies on SII are very scarce, except for one that characterized SII as possessing three disulfide bonds and no net positive charge [32].

### 2.2. α4/3 Conotoxins

Asp 5, Pro 6, Arg 7, and Trp 10 are critical residues of the ImI conotoxin, conferring its specificity for the α7 nAChRs [33].

Despite the fact that ImI and ImII conotoxins share a high sequence homology (9 of 12 amino acids are identical) (Figure 2), they were found to target different sites at the α7 nAChR, since only ImI (and not ImII) showed competitive inhibition of the receptor when α-bungarotoxin (α-Bgtx) was co-added [34]. Mutational studies revealed that the amino acid residue at position 6 (Pro in α-Ctx ImI, Arg in α-Ctx ImII) determines the selectivity for a specific site, whereas the amino acid residue in position 9 (Ala in α-Ctx ImI, Arg in α-Ctx ImII) confers each of the toxins its optimal affinity to bind to their corresponding sites at the α7 nAChR [34].

The selectivity and potency of α-conotoxin RgIA for the α9α10 subtype of nAChR is conferred by Arg 9 and Arg 13 residues [35]. It is also interesting to note that [3,12]-dicarba RgIA is able to act specifically at α9α10 nAChRs, losing its effect on N-type calcium channels [36]. This analogue could potentially be used in a clinical trial for eliciting an effect on a single receptor subtype.

### 2.3. α4/4 Conotoxins

α-conotoxin BuIA blocks α6/α3β2 nAChRs with a significantly lower IC_50_ than α4β2 receptors [37]. In fact, there is a 40,000-fold difference between their IC_50_ values [37]. The five residues of the α6 subunit, Lys 85, Asp 187, Ile 188, Thr 198, and Tyr 205, were determined to be responsible for this effect [38].

It should be noted that α-conotoxin BuIA on nAChRs with β4 subunits have slow off-time when compared to corresponding nAChRs with β2 subunits [37]. This implies that BuIA can discriminate between different β subunits based on the time needed to unblock the receptor.

The α-conotoxin BuIA [T5A; P6O] was found to be selective for α6β4 vs. α6β2 nAChRs [39]. It was mainly achieved by P6O substitution, which resulted in a 2800-fold increase of IC_50_ at α6/α3β2β3 and only a 6-fold increase at α6/α3β4 [39].

It is interesting to note that, though α-conotoxins EIIA and PIB come from different *Conus* species, they are almost identical in sequence, except for one residue at the second position. Therefore, it is not surprising that they both have selectivity for muscle-type nAChRs [40]. This could be due to Asn 4, Pro 5, Ala 6, and Lys 9 residues located in the first and second loops, respectively, which were previously found to be critical for the muscle nAChR selectivity of α3/5 conotoxins [40].

### 2.4. α4/6 Conotoxins

Alanine scanning mutagenesis revealed three residues in the AuIB conotoxin (Gly 1, Pro 6, and Phe 9) that affect the inhibition of α3β4 nAChRs [41]. Gly 1 participates in the formation of a salt bridge between the N-terminus of the peptide and Asp 72 of the β4 subunit. Hence, its substitution with alanine disrupted a favorable interaction of AuIB with the receptor. However, most of the inhibitory effect is elicited particularly by Pro 6 and Phe 9. It was elucidated that Pro 6 caused inhibition due to its effect on 3D structure of the peptide, whereas Phe 9 was responsible for the interaction with a two-residue binding pocket (Trp 59 and Lys 61) of the β4 subunit [41].

ViIA is a unique α-conotoxin that specifically targets the α3β2 nAChR subtype [42]. Structure–activity studies on the ViIA conotoxin revealed that Arg 1 and His 11 are critical residues for binding to the target effectively. Interestingly, a mutant ViIA[+16L] (a mutant with additional Leu residue at position 16) produced a 12-fold stronger interaction with α3β2 nAChR when compared to the native peptide. This was explained by hydrophobicity Leu residue conferred to the overall structure of the ViIA conotoxin [42].

The VnIB conotoxin selectively inhibits α6β4 nAChRs [43]. However, no structure–activity studies on this conopeptide have been carried out so far. It is hypothesized that all of the members of α4/6 conotoxins, including VnIB, exhibit β4 subunit selectivity due to their common 4/6 substructure. The selectivity of VnIB toward α6-containing nAChRs can potentially be explained by the five residues present in the second disulfide-loop, excluding Pro residue (YTKNPN) [43].

There are five residues (His 5, Pro 6, Val 7, Met 11, and Pro 13) of TxID critical for the inhibition of α3β4 and α6/α3β4 nAChRs [44]. [S9A] TxID mutant was found to discriminate between the two receptor subtypes, having a 46-fold higher affinity to α3β4 than to α6/α3β4 nAChRs [44].

### 2.5. α4/7 Conotoxins

Ala 11 and Gln 14 play a role in α3β2 selectivity of AnIB, whereas the C-terminal amidation and sulfation of Tyr 16 are responsible for α7 nAChR binding [45].

ArIB [V11L; V16D] is considered to be the most selective α7 nAChR antagonist to date (IC_50_ = 1.09 nM) [46].

Important residues conferring EI potency to bind mouse α1β1δε nAChR were found to be His 7, Pro 8, Met 12, and Pro 15. In addition, deletion of Arg1–Asn2–Hyp3 residues are also crucial, since their deletion causes a total loss of activity at muscle receptors [47]. [S13A] EI mutant exhibited increased potency and selectivity for α1β1δε nAChRs [47].

GIC is a very interesting conotoxin from a research standpoint, since it exhibits extreme potency and selectivity towards α3β2 nAChRs (IC_50_ = 1.1 nM) [48]. His 5 and Gln 13 were found to be the most important residues that confer GIC its potency and selectivity, respectively. In addition, Ala 7, Asn 11, and Asn 12 residues of the peptide were involved in binding to the principal site of the receptor [48].

Ala 10, Val 13 and Val 18 are important residues of the GID conotoxin, conferring it selectivity for α4β2 over α3β2 nAChRs [49]. In particular, GID[V18N] analog exhibits the highest selectivity for α4β2, with no inhibitory effect on the α3β2 subtype [49].

The structure–functional study conducted to the Lo1a conotoxin found that its Asp-18 residue is critical for the selectivity of the peptide for neuronal vs. muscle subtype nAChRs [50]. This was evident from the observation that Lo1a-ΔD and Lo1a-RRR analogues, which either lacked Asp 18 or had it replaced with 3 Arg residues, respectively, acquired affinity for the adult muscle subtype α1β1δε, aside from α7 receptors [50].

Ser-1 and Gly 2 are important residues for the affinity of LsIA to α3β2 and α7 nAChRs [51]. In addition, carboxylation of the C-terminus of LsIA resulted in its selectivity for α3β2 over α7 to increase 9-fold [51].

Selectivity of the Lt1.3 conotoxin for α3β2 receptors is attributed to a small hydrophobic Gly 10 residue, whereas Asn 11, Asn 12, Pro 13, Tyr 14, and Phe 15 residues contribute to the receptor binding [52].

His 5, Pro 6, Ala 7, and His 12 of LvIA are important residues that interact with α3-containing nAChRs [53]. LvIA exhibits unusual selectivity toward α3β2 vs. α6/α3β2β3 nAChRs due to Asn 9 and Asp 11 residues [54].

Asp 5, Pro 6, and His 12 are the main residues of the MII conotoxin, which confers its potency for binding to the rat α3β2 nAChR subtype [55]. The MII [H9A; L15A] analog of α-conotoxin MII is selective towards α6-containing receptors (IC_50_ on rat α6/α3β2β3 = 2.4 nM), and can discriminate between α6 and α3 subunits [56].

Mr1.7 [E2A] is potent and selective for the α3β2 nAChR subtype, with no inhibitory effect on other subtypes [57]. However, the substitution of Ser 12 with Ala results in the loss of selectivity to α3β2, with new binding ability to other subtypes (α3β4, α2β4, and α7) [57].

The PeIA conotoxin can be made >15,000-fold more potent on α6/α3β2β3 vs. α3β2 nAChRs via substituting Asn 11 with either Lys or Arg [58]. On the contrary, PeIA-4566, which is a synthetic peptide composed of non-natural amino acids, targets α3β2 over α6/α3β2β3, with a 300-fold difference in potency [59]. PeIA [S9H; V10A; E14N] analog blocks α6/α3β2β3 and α6/α3β4 nicotinic receptors with IC_50_ values of 223 pM and 65 nM, respectively [60]. Thus, it can be concluded that PeIA [S9H; V10A; E14N] discriminates between α6β2 and α6β4 nAChRs.

Residues from Leu 5 to Pro 13 and Tyr 15 are important residues of PnIA, conferring its inhibitory effect on α7 nAChRs [61]. In addition, the PnIA [A10L] mutant is able to exclusively bind α7 nAChRs [62].

The RegIIA [N11A; N12A] analog selectively blocks α3β4 vs. α3β2 nAChRs, with a 1000-fold difference in potency [63].

TxIB is unique due to its ability to selectively bind α6/α3β2β3 nAChRs, while lacking any capability to bind to other receptor subtypes [64]. However, structure–functional studies accounting for this have not been conducted yet.

It is generally accepted that α-Ctxs are antagonists of nAChRs, but MrIC (PECCTHPACHVSNPELC), which is a peptide variant of Mr1.7, plays a full agonist role of endogenous human α7 nAChRs in the presence of PNU (positive allosteric modulator of α7 nAChR subtype) [65]. It is suggested that the Pro residue in the N-terminus of the peptide has a role in binding α7 nAChR [65].

It was found that Asp 5 to Arg 7, and Asp 11 to Ile 15, are critical residues for the inhibitory effect of Vc1.1 at the α9α10 nAChR subtype [66]. In addition, mutations at positions 4 and 9 (substitution of Ser 4 with either Lys or Arg and substitution of Asn 9 with either Ala, Leu, or Ile) increased the potency of Vc1.1 at α9α10 nAChRs [66].

### 2.6. Therapeutic Potential of α-Conotoxins

Due to the fact that nAChRs are implicated in a range of pathological conditions, α-conotoxins targeting nAChRs pose themselves as attractive candidates for drug development [67,68,69]. For example, α-conotoxins RgIA and Vc1.1 demonstrate the analgesic effect in animal models of neuropathic pain [14,70,71,72,73,74,75,76]. Such analgesic effect works through targeting α9α10 nAChRs. The mechanism of analgesia mediated by RgIA and Vc1.1 conotoxins is not currently clear, but there are indications that it involves immune-mediated pathways.

RgIA was shown to be potent in reducing mechanical allodynia and oxaliplatin-induced hypersensitivity to thermal and mechanical stimuli [77], as well as accelerating the recovery of nerve damage following chronic constriction injury (CCI) [70]. Other than alleviating neuropathic pain, RgIA was also shown to be able to reduce the severity of dextran sodium sulfate-induced colitis in an animal model [78].

Vc1.1 was shown to reduce mechanical allodynia in rats with diabetic neuropathy induced by streptozotocin, and in rats with CCI [14,74]. Vc1.1 reduced mechanical hyperalgesia in rats with peripheral nerve ligation when the toxin was injected intramuscularly [75].

Another α-conotoxin with a potential therapeutic application due to its antitumor activity is TxID. The toxin inhibited the growth of lung cancer cells [79].

The results from in vitro and animal studies serve as an encouragement for drug development. There are, however, hindrances along the way [80], which stem from the peptidic nature of the toxins, the potential off-target effects, and different potencies on human receptors vs. rodent ones. For example, Vc1.1 was discontinued from phase II human clinical trials due to its low affinity on human α9α10 nAChRs [15] (IC_50_ on rat α9α10 is 20–100 nM, on human α9α10 is 1 μM).

**Table 1:** The α-conotoxins mentioned in the main text are shown, together with their targets and potencies.

α-Conotoxin	Species	Target and IC_50_ / Ki	Reference	UniProt/PDB
Ac1.1a	*C. achatinus*	m(α1)2β1δγ (IC_50_ = 36 nM)	[27]	P0CAQ4/n.d.
Ac1.1b	*C. achatinus*	m(α1)2β1δγ (IC_50_ = 26 nM)	[27]	P0CAQ5/n.d.
CIA	*C. catus*	r(α1)2β1δγ (IC_50_ = 5.7 nM) rα3β2 (IC_50_ = 2.06 μM)	[31]	D4HPD6/n.d.
GI	*C. geographus*	*Torpedo californica* (IC_50_ α/γ site = 4.5 nM) (IC_50_ α/δ site = 87 nM) m(α1)2β1δγ (IC_50_ α/δ site = 1.3 nM) (IC_50_ α/γ site = 60 μM) rα9α10 (IC_50_ = 9.35 μM)	[29,30]	P01519/1XGA
MI	*C. magus*	m(α1)2β1δγ (IC_50_ = 0.4 nM)	[26]	P01521/n.d.
SIA	*C. striatus*	m(α1)2β1δγ (IC_50_ = 2.6 nM) (IC_50_ = 2.3 μM)	[28]	P28878/n.d.
SII	*C. striatus*	m(α1)2β1δγ (IC_50_ = 18 μM)	[28]	P28879/6OTB
ImI	*C.imperialis*	hα3β2 (IC_50_ = 40.8 nM) hα3β4 (IC_50_ = 3.39 μM) hα7 (IC_50_ = 595 nM) rα7 (IC_50_ = 191 nM) rα7 (IC_50_ = 69.3 nM)	[34,81]	P50983/1CNL
ImII	*C.imperialis*	h(α1)2β1δε (IC_50_ = 1.06 μM) hα3β2 (IC_50_ = 9.61 μM) hα7 (IC_50_ = 571 nM) rα7 (IC_50_ = 441 nM)	[34,81]	Q8I6R5/n.d.
RgIA	*C. regius*	rα9α10 (IC_50_ = 4.55–5.19 nM) rα7 (IC_50_ = 4.66 μM)	[82]	P0C1D0/2JUT
BuIA	*C. bullatus*	rα6/α3β2 (IC_50_ = 0.3 nM) rα6/α3β2β3 (IC_50_ = 0.46 nM) rα6/α3β4 (IC_50_ = 1.5–2.1 nM) rα2β4 (IC_50_ = 121 nM) rα3β2 (IC_50_ = 5.7 nM) rα3β4 (IC_50_ = 28 nM) rα4β4 (IC_50_ = 70 nM) rα7 (IC_50_ = 272 nM)	[37,39]	P69657/2I28
EIIA	*C. ermineus*	*Torpedo* (Ki = 0.46–105 nM) α7-5HT3 (Ki >>1000 nM) α3β2 (Ki >>1000 nM) α4β2 (Ki >>1000 nM)	[40]	D4HRK4/n.d.
PIB	*C. purpurascens*	m(α1)2β1δε (IC_50_ = 36 nM) m(α1)2β1δγ (IC_50_ = 45 nM)	[83]	P0C351/n.d.
AuIB	*C. aulicus*	α3β2/α3β4 (intracardiac ganglia) (IC_50_ = 1.2 nM) rα3β4 (recombinant) (IC_50_ = 750–966 nM)	[84,85]	P56640/1MXN
ViIA	*C. virgo*	rα3β2 (IC_50_ = 845.5 nM)	[42]	F5C0A0/n.d.
VnIB	*C. ventricosus*	rα6β4 (IC_50_ = 12 nM) rα6/α3β4 (IC_50_ = 18 nM) rα3β4 (IC_50_ = 320 nM) rα6/α3β2β3 (IC_50_ = 4000 nM) hα6/α3β4 (IC_50_ = 5.3 nM)	[43]	A0A4P8XV20/n.d.
TxID	*C. textile*	rα3β4 (IC_50_ = 12.5 nM) rα6/α3β4 (IC_50_ = 94 nM)	[64]	K8DWB5/n.d.
AnIB	*C. anemone*	rα3β2 (IC_50_ = 0.3 nM) rα7 (IC_50_ = 76 nM)	[45]	P0C1V7/n.d.
ArIA	*C. arenatus*	rα7 (IC_50_ = 6 nM) rα3β2 (IC_50_ = 18 nM)	[46]	P0C8R2/n.d.
ArIB	*C. arenatus*	rα7 (IC_50_ = 1.8 nM) rα6/α3β2β3 (IC_50_ = 6.45 nM) rα3β2 (IC_50_ = 60.1 nM)	[46]	P0C8R2/n.d.
CIB	*C.catus*	rα3β2 (IC_50_ = 128.9 nM) rα7 (IC_50_ = 1.51 μM)	[24]	P0DPT2/n.d.
EI	*C. ermineus*	m(α1)2β1δε (IC_50(1)_ = 9.4 nM) (IC_50(2)_ = 280 nM) *Torpedo* (IC_50(1)_ = 0.41 nM) (IC_50(2)_ = 190 nM)	[86]	P50982/1K64
GIC	*C. geographus*	hα3β2 (IC_50_ = 1.1 nM) hα3β4 (IC_50_ = 755 nM) hα4β2 (IC_50_ = 309 nM)	[87]	Q86RB2/1UL2
GID	*C. geographus*	rα3β2 (IC_50_ = 3.1 nM) rα7 (IC_50_ = 4.5 nM) rα4β2 (IC_50_ = 152 nM)	[88,89]	P60274/1MTQ
Lo1a	*C. longurionis*	α7 (IC_50_ = 3.24 μM)	[50]	X1WB75/2MD6
LsIA	*C. limpusi*	α3β2 (IC_50_ = 10 nM) α3α5β2 (IC_50_ = 31 nM) α7 (IC_50_ = 10 nM)	[51]	P0DL68/n.d.
LtIA	*C. litteratus*	α3β2 (IC_50_ = 9.8 nM) α6/α3β2β3 (IC_50_ = 84.4 nM) α6/α3β4 (IC_50_ = 6 μM)	[90]	Q2I2R8/n.d.
Lt1.3	*C. litteratus*	rα3β2 (IC_50_ = 44.8 nM)	[52]	n.d.
LvIA	*C. lividus*	α3β2 (IC_50_ = 8.7 nM) α6/α3β2β3 (IC_50_ = 108 nM) α6/α3β4 (IC_50_ = 121 nM) α3β4 (IC_50_ = 148 nM) α7 (IC_50_ = 3 μM) hα3β2 (IC_50_ = 17.5 nM) hα6/α3β2β3 (IC_50_ = 5.34 μM)	[53]	L8BU87/2MDQ
MII	*C. magus*	α6/α3β2β3 (IC_50_ = 0.4 nM) α3β2 (IC_50_ = 0.5/3.7 nM)	[55,56,91]	P56636/1M2C
Mr1.7	*C. marmoreus*	rα3β2 (IC_50_ = 53.1 nM) rα9α10 (IC_50_ = 185.7 nM) rα6/α3β2β3 (IC_50_ = 284.2 nM)	[57]	F6LPN3/n.d.
PeIA	*C. pergrandis*	rα9/α10 (IC_50_ = 6.9 nM) rα7 (IC_50_ = 1.8 μM) rα3β2 (IC_50_ = 23 nM) rα3β4 (IC_50_ = 480 nM)	[92]	Q1L777/n.d.
PnIA	*C. pennaceus*	rα3β2 (IC_50_ = 9.56 nM) rα7 (IC_50_ = 252 nM)	[61]	P50984/1PEN
RegIIA	*C. regius*	rα3β4 (IC_50_ = 97 nM) rα3β2 (IC_50_ = 33 nM) hα7 (IC_50_ = 103 nM)	[93]	P85013/n.d.
TxIB	*C. textile*	rα6/α3β2β3 (IC_50_ = 28 nM)	[64]	K4RNX9/2LZ5
Vc1.1	*C. victoriae*	rα9α10 (IC_50_ = 109 nM) hα9α10 (IC_50_ = 549 nM) rα6/α3β2β3 (IC_50_ = 140 nM) rα3β4 (IC_50_ = 4.2 μM) rα3β2 (IC_50_ = 7.3 μM)	[66,94]	P69747/2H8S

## 3. Snake Venoms as a Source of Toxins Acting on Acetylcholine Receptors

Snakes are a rich source of ligands targeting nAChRs. The neurotoxins of snake origin were characterized long ago and include α-Bgtx from *Bungarus meltinctus* and α-cobratoxin (α-Cbtx) from *Naja naja siamensis*. The biologically active toxins in snake venoms can be organized into molecules with enzymatic or without enzymatic activities. Many snake neurotoxins of non-enzymatic action possess a conserved structural feature consisting of three-finger loops, where β-sheet fingers protrude from a globular hydrophobic core coordinated by disulfide bonds [95]. Neurotoxins targeting postsynaptic nAChRs are called α-neurotoxins (α-NTX) [96]. Depending on the length and number of disulfide bridges, three-finger α-neurotoxins are divided into short-chain toxins (type I) and long-chain toxins (type II). Long-chain toxins contain 66–75 amino-acids and ten cysteines, whereas short-chain toxins have 60–62 amino-acids and eight cysteines. Other than three-fingered neurotoxins with a typical structure, more and more toxins with alternate structures are being discovered. For example, non-conventional three-finger neurotoxins are similar to long-chain α-NTX in their length and number of disulfide bonds; however, the fifth disulfide bond is located within finger I of the toxin, unlike in finger II within long-chain α-NTX [97]. The toxins within this group include “weak toxin” WTX [98] and candoxin [99]. Some snake toxins can form homo- or heterodimers. Homodimers of short-chain three-finger α-NTX include fulditoxin and haditoxin. Homodimers of long-chain α-NTX include the α-cobratoxin dimer [100,101]. Homodimers can be formed as a result of covalent interactions, as in α-cobratoxin, or noncovalent interactions, as in κ-bungarotoxin, fulditoxin, and haditoxin [101,102]. Heterodimeric toxins include irditoxin with intermolecular disulfide bonds [103].

### 3.1. Long-Chain Three-Finger α-NTXs

Long-chain three-finger α-NTs interact with muscle and neuronal nAChRs. To the neuronal α7 receptor, these toxins bind with high affinity, Kd values at 10^−9^ to 10^−8^ M [104]. The residues within finger II of the toxin were shown to be important for binding to this receptor type. The mode of interaction of two long-chain three-finger α-NTXs, α-bungarotoxin and α-cobratoxin, will be considered below.

α-bungarotoxin contains 74 amino-acids and 5 disulfide bonds. The toxin was isolated in 1963 by Chang and Lee [105] from a Taiwan banded krait *Bungarus multicinctus*, and helped in the characterization of muscle-type nAChRs [106]. Original discovery of the toxin demonstrated its paralytic effect in rodent muscle preparation, and later it was shown that the toxin targets muscle nAChRs with high affinity (Kd ~ 5 nM), acting as a competitive antagonist in an irreversible fashion [104]. The toxin also blocks *Torpedo* acetylcholine receptor in the irreversible fashion [107]. Besides targeting muscle-type receptor, it also has high affinity towards homomeric neuronal receptors, such as α7 and α9, with nanomolar affinity [108]. An NMR structure of the toxin (PDB: 1KFH) shows three fingers projecting from the globular core, shaped by four disulfide bonds, with an additional disulfide bond within finger II. Structure–functional studies revealed significance of cation-π interaction in stabilizing the toxin within the ligand-binding site. Arg 36 plays a major role in toxin binding to muscle receptor type, as well as for neuronal receptor type [109]. This is due to similarities in the structure of guanidinium group within Arg 36 and quaternary ammonium of acetycholine, which directs toxins into the agonist binding site.

Multiple crystal structures of the toxin bound to its target were solved, which helped in understanding of the mode of toxin–ligand interaction by revealing the orientation of the toxin within binding site of the receptor. Moreover, 1.94 Å crystal structure of α-Bgtx bound to extracellular domain of α1 subunit of mouse muscle receptor is available (PDB: 2QC1) [110], which reveals the *N*-glycosylated Asn 141 on the receptor side, with oligosaccharide composed of two *N*-acetylglucosamines (GlcNAc 2–3) and eight mannoses (Man 4–11), and forming hydrogen bonds with the toxin. The study demonstrated the orientation of the toxin within an aromatic cage of the ligand-binding interface. Finger I is surrounded by loop C and carbohydrate; the tip of finger II of the toxin is oriented into the ligand-binding site made up by loops A, B, and C on the receptor side, and finger III is away from the receptor. Arg 36 and Phe 32 within finger II on the toxin side form a cation-π stack and oppose Tyr 198 from the receptor side in a “face-to-face” fashion. Two other tyrosines on the receptor side, Tyr 190 and Tyr 93, are also oriented towards Arg 36 and Phe 32, but face their edges. The structure also revealed hydrogen bonds between Arg 36, Thr 148, and Cys 192. Despite the wealth of information obtained from this crystal structure, the lack of adjacent subunit obscures the entire picture. Additionally, Trp 149 to Arg substitution, which was performed for crystallization purposes, removed Trp, which is important for toxin–receptor interaction.

The crystal structure of the α-Bgtx bound to human α7–AChBP chimera resolved to 3.5 Å provided insight into the toxin interaction with adjacent subunits within a ligand-binding interface (PDB: 4HQP), where loop C of the receptor made contact with finger II of the toxin [111]. Particularly, Tyr 184, which is equivalent to Tyr 190 in mouse α1 subunit, is oriented towards the edge of cation-π stack, formed by Arg 36 and Phe 32. This is analogous to the earlier crystal structure. Tyr 184 is itself hydrogen-bonded through hydroxyl-group, with Asp 30 of the α-Bgtx. Other residues from loop A (Tyr 91), loop B (Trp 145), and loop C (Tyr 191, Arg 182) also contribute to the toxin binding.

The crystal structure of α-Bgtx complexed with the extracellular domain of α9 subunit at 2.7 Å was solved (PDB: 4UY2), and it revealed the mode of interaction between the toxin and a principal subunit of the receptor. Thus, Arg 36 and Phe 32 of finger II of the α-Bgtx are positioned deeply in the ligand-binding site of the α9 subunit, where they interact with residues of loops A, B, and C. Particularly, Arg 36 makes a cation-π interaction and hydrogen bond with Tyr 95. Asp 30 forms a hydrogen bond with Tyr 192. Finger I interacts with loop C, with Val 40 forming a hydrogen bond with Ser 191. Finger III is oriented away from the ligand-binding site. Due to the fact that only a principle subunit interface is revealed by the structure, the contribution of the complementary subunit is not seen [112].

The Cryo-EM structure of α-Bgtx complexed with native receptor from Torpedo electric organ (PDB: 6UWZ) revealed details of the toxin–receptor interaction [113] with 2.7 Å resolution. The structure revealed residues on α-δ and α-γ interfaces important for binding. The study highlighted the involvement of residues within complementary side on the ligand-binding interface, particularly within loop F. The structure showed the penetration of finger II of the toxin deep into the ACh binding pocket, where it makes contact with Tyr 93, Tyr 190, and Tyr 198 of the α subunit. Similar to the previous studies, Arg 36 forms a cation-π interaction with Tyr 198 of the principal receptor subunit and Phe 32 of the toxin.

α-cobratoxin is a 71 amino-acid-long toxin from the Indo-Chinese spitting cobra *Naja naja siamensis* which demonstrates curarimimetic activity. Residues important for binding to muscle-type receptor include Lys 23, Trp 25, Asp 27, Phe 29, Arg 33, and Lys 49. In addition, mutational studies revealed the importance of Arg 36 and Phe 65 for binding to α7 nAChRs and the *Torpedo* receptor [114], with some residues specific for binding to the α7 receptor (Ala 28, Lys 35, Cys 26, and 30), while others were specific for binding to the *Torpedo* receptor (Lys 23, Lys 49). The double-mutant cycle analysis for α-Cbtx interaction with the α receptor revealed Phe 29–Trp 54 coupling [115]. The crystal structure of α-cobratoxin complexed with AChBP from *Lymnaea stagnalis* [116] revealed more details of a toxin–receptor interaction, where the toxin inserts its finger II deep into the ligand-binding pocket, with Phe29 and Arg33 of the toxin mimicking acetylcholine at the interface between two subunits. Phe29 and Arg33 engage in hydrophobic and aromatic interactions with AChBP Trp53, Tyr185, Tyr192, and Trp143. Additionally, Trp25, Asp27, Ala28, and Ile32 are close to Tyr185 in the principle subunit interface of AChBP and Glu163, Glu55, Leu112, Met114, and Tyr164 in a neighboring complementary subunit. Possible interactions could involve Ser31, Cys26, and Cys30 within finger II of the toxin, and AChBP Ser159–Tyr164 in a complementary subunit on the receptor side. The toxin displaces loop C of the subunit.

αδ-bungarotoxin-1 is a 73 amino-acid-long toxin isolated from *Bungarus candidus*, with five disulfide bonds and inhibitory activity in muscle and neuronal α7 nAChRs. Unlike α-Bgtx, αδ-Bgtx binds to the target receptor in a reversible manner. Binding affinities are higher at the α-δ interface than they are at the α-γ or α-ε interfaces [117]. The reason for such selectivity could be due to the shorter finger I, and thus a smaller number of contacts with the receptor interface, as well as fewer positively charged amino acids within finger II of the toxin.

Drysdalin is a long-chain NTX with 87 amino acids. It has five disulfide bonds and possesses curarimimetic activity with the irreversible inhibition of muscle receptors. The toxin demonstrates nanomolar affinity towards rodent muscle (α1)2β1δε, as well as human α7 and α9α10 nAChRs. Arg 30 and a 24-residues-long C-terminal tail were shown to be important for toxin binding. The toxin has a 24-long C-terminus tail, the truncation of which abolishes the activity of the toxin on α9α10 nAChRs [118]. The toxin has a highly divergent structure within the second finger, where three of eight highly conserved in long-chain NTX residues, important for receptor interaction, are altered. Despite the lack of conserved residues within the second finger, the toxin retains properties of the long-chain three-finger α-NTXs, and causes irreversible postsynaptic neurotoxicity at a nanomolar concentration (IC_50_ on chick biventer cervices muscle preparation is 38.7 nM). Despite the sequence difference, the toxin retains the target specificity of the long-chain α-NTXs, suggesting functional rather than structural similarity between the long-chain toxins

### 3.2. Short-Chain Three-Finger α-Neurotoxins

Erabutoxin a and b are 62 amino-acid-long short-chain neurotoxins isolated from the Chinese sea snake *Laticauda semifasciata* with four disulfide bonds. The toxins are potent inhibitors of the muscle-type receptor, *Torpedo* receptor, and is a typical curarimetic toxin [119]. The toxin is a weak antagonist of homomeric neuronal α7 nAChRs. Mutagenesis studies revealed a contribution of the conserved core residues within finger II, such as Lys 27, Trp 29, Asp 31, Phe 32, and Arg 33 for receptor binding [120,121]. In addition, His 6, Gln 7, Ser 8, and Gln 10 of finger I, as well as additional residues of finger II, such as Tyr 25, Gly 34, Ile 36, and Clu 38, are important for receptor binding.

NmmI is a 62 amino-acid long short-chain neurotoxin isolated from *Naja mosambica* possessing high affinity towards muscle-type nAChR. The neurotoxin is capable of distinguishing α-γ and α-δ subunit interfaces from an α-ε interface; it has an affinity 3 orders of magnitude higher towards the former compared to the latter [122,123]. Mutational studies revealed the significance of Lys 27 for toxin binding, and double-mutant cycle analysis revealed the interaction between Lys 27 and Glu 176 on γ subunit [123].

### 3.3. Dimeric Toxins

Dimeric toxins demonstrate changes in target specificity compared to a monomeric form, with many dimeric toxins targeting neuronal types of nAChRs. Fulditoxin, haditoxin, dimeric α-cobratoxin, κ-bungarotoxin, and irditoxin are examples of dimeric toxins. Fulditoxin is a homodimer of two short-chain three finger α-NT, with monomers linked together through 29 hydrophobic interactions between residues within Finger II of the monomer [102]. Haditoxin is a homodimer of two short-chain α-3FNTX, with monomers bound to each other through 14 hydrogen bonds between finger III residues [124]. α-cobratoxin dimer contains two covalently joined monomers bound by two disulfide bonds [100,101]. *Κ*-bungarotoxin is a dimeric NTX, where two identical monomers are bound through 9 hydrogen bonds [100]. The dimeric nature of the toxin changes its binding properties, making the toxin lose its potency on muscle receptors while enabling its interaction with neuronal types of receptors (α3β2, α7, and α4β2 nAChRs). Irditoxin is a heterodimeric toxin with two protomers covalently bound by a disulfide bond. The toxin demonstrates specific activity on avian muscle receptors, with a 1000-times higher activity on avian receptors compared to rodent receptors (10 nM vs. >10 μM IC_50_) [103] (Table 2).

### 3.4. Non-Conventional Toxins

Weak toxin (WTX) isolated from cobra *Naja kaouthia* is a 65 amino-acid-long toxin with five disulfide bonds. By length, it is similar to short-chain toxins; by number of disulfide bonds, it is similar to long-chain toxins. However, unlike in long-chain neurotoxins, the fifth bond is within the first finger. The toxin is named “weak” because of its low toxicity. It was reported that, at 2 mg/kg delivered intravenously, it was not toxic on rats [98]. The toxin inhibits *Torpedo* nAChR with a Kd of 90 nM, and it also shows affinity towards neuronal α7 and muscarinic receptors present within adrenal medulla cells [130]. Arg 31 and Arg 32, both within the second finger, are important for the interaction with muscle and α7 nAChRs [131]. The same residues, together with Arg 37, are crucial for binding to human muscarinic M1 and M3 receptors [139].

Candoxin is a 66-amino-acid-long toxin isolated from *Bungarus candidus* snake, similar in length to short-chain toxins. However, unlike short-chain toxins, it has five disulfide bonds. The location of the fifth bond is within finger I, which is similar to WTX [97,99]. Candoxin blocks muscle and neuronal α7 nAChR. IC_50_ on rat α1β1δγ expressed in oocytes is 10 nM, on rat α7 is 50 nM. The inhibition by candoxin is reversible for muscle-type of receptors, and poorly reversible for α7 nAChR. LD_50_ of candoxin is 0.83 mg/kg, which is higher than of WTX [97].

### 3.5. Toxins with Unique Structure

Denmotoxin is a 77-amino-acid-long toxin isolated from *Boiga dendrophila* snake, with a unique property to discriminate between its targets. It irreversibly blocks chick αβδγ nAChR, while it is a 100-fold weaker antagonist of mouse muscle nAChR [132]. Injected intraperitoneally to mice at 20 mg/kg, no biological effect was observed. Structurally, it possesses five disulfide bonds, with an extra disulfide bond within loop I of the toxin.

Lc-a and Lc-b are both 69 amino-acid-long toxins isolated from sea snake *Laticauda colubrina* [133]. They both contain four disulfide bonds and are lethal when injected intramuscularly to mice with an LD_50_ of 0.12 mg/kg.

Ω-Neurotoxin Oh-9 is a 57 amino-acid-long toxin isolated from *Ophiophagus Hannah*, with four disulfide bonds and a low sequence similarity with α-neurotoxins of snake origin [134]. The toxin inhibits rat muscle α1β1εδ nAChRs, as well as α3β2 receptor subtype. Mutagenesis studies revealed that the tip of loop II is not crucial for binding. Met 25 and Phe 27 constitute the binding core of the toxin to both muscle and neuronal receptor types, whereas Thr 23 and Phe 26 are critical for interaction with α1β1εδ but not with α3β2 [135].

αδ-bungarotoxin-1 is 73 amino-acid-long toxin isolated from *Bungarus candidus,* with five disulfide bonds and inhibitory activity on muscle and neuronal α7 nAChRs. Unlike α-Bgtx, αδ-Bgtx binds to the target receptor in a reversible manner. Binding affinities are higher at the α-δ interface than at the α-γ or α-ε interfaces [117]. The reason for such selectivity could be due to a shorter finger I, and thus a smaller number of contacts with the receptor interface, as well fewer positively charged amino acids within finger II of the toxin.

Waglerin-1 is 22 amino-acid-long peptide with a single disulfide bond. Despite its difference in length and structural features with other snake neurotoxins, Waglerin-1 blocks the muscle nAChR containing ε subunit [136,140], and has a 2000-fold higher affinity for α-ε compared to the α-δ subunit interface. The toxin is not potent on the fetal muscle receptor containing the γ subunit [136]. Besides its activity on nAChRs, Waglerin-1 also potentiates and suppresses GABA_A_ receptors (EC_50_ = 24 μM) [137].

## 4. Peptides of Spider Venom Origin as Potential Ligands Acting on nAChRs

Spiders do not possess a diverse arsenal of toxins which act on acetylcholine receptors. This fact is surprising when considering the importance of acetylcholine receptors for insects, the main prey of spiders. The action of spider toxins on nicotinic receptors is observed, alongside their activity on other targets.

The toxin HWTX-1 (Huwentoxin-1), from a Chinese bird spider *Selenocosmia huwena* (synonymous to *Haplopelma schmidti*) [141,142], is a 33 amino-acid-long peptide with six cysteines connected in a following way: C1–C4; C2–C5; C3–C6, which is similar to disulfide bonds of ω-conotoxin GVIA. HWTX-1 was initially shown to be potent in inhibiting neuromuscular transmission and evoking muscle paralysis. At 10 ug/mL, the blockade of twitch response was achieved in 15.2 min in a mouse phrenic nerve-hemidiagraphm preparation [142]. Later, however, the effect of the toxin on acetylcholine receptors was questioned [143,144,145]. Now, it is established that the main target of the HWTX-I is the N-type Calcium channel (IC_50_ is 100 nM) [144].

Another toxin of spider origin with potential activity on nicotinic acetylcholine receptors is ω-Agatoxin IVA, derived from the American funnel spider, *Agelenopsis aperta*. The inhibitory activity of the toxin was demonstrated on bovine chromaffin cells, where the toxin at a 100 nM concentration inhibited nicotine-evoked current [146]. However, besides this, the toxin was not shown to be active on nicotinic receptors. The main mechanism of its action is through the blockage of P-type voltage-gated calcium channels [147,148].

The only peptidic toxin of spider origin that was shown to affect nicotinic receptors is κ-Hexatoxin Hv1c (κ-HXTX-Hv1C), from the blue-mountain funnel web spider, *Hadronyche versuta* [149]. It acts as a positive allosteric modulator of the receptors (Table 3).

## 5. Peptides of Scorpion Venom Origin as Potential Ligands Acting on nAChRs

Scorpion venom is a rich source of ligands targeting voltage-gated potassium (Kv) and sodium (Nav) channels [150,151]. The affinity for these channels is at nM and even at pM range. Scorpion toxins are classified into several families and subfamilies based on four criteria. The criteria include the type of affected ion channel (sodium, potassium, calcium, or chloride) [152], target site on the channel, three-dimensional structure, and type of response. The Nav-targeting toxins are divided into α- and β- type depending on the binding site on the channels and the resultant effect of binding. α-toxins bind at site 3 and inhibit channel inactivation, while β- toxins bind at site 4 and change channel activation. The Kv-targeting toxins are divided into α-, β-, γ-, and κ- families. κ- family toxins have α-helix-loop-helix fold, where two short α-helices are connected by a β-turn [152].

A recent report by Kasheverov [153] showed that scorpion peptides demonstrate target promiscuity (Table 4). Thus, scorpion toxins within α-KTx and κ-KTX families target nicotinic AChRs of muscle and α7 type, with a higher affinity towards muscle-type receptor vs. homomeric neuronal α7 type. For example, the IC50 of HelaTx1 on *Torpedo californica* nAChRs is 1.4 ± 0.1 μM; on human, α7 nAChR is 64 ± 7 μM (Table 4). HelaTx 1 is a toxin within the κ-KTx family with two short α-helices stabilized by two disulfide bonds; by structure, it resembles α-conotoxins. Surprisingly, it has a higher affinity towards muscle-type nAChRs compared to the Kv channel. Overall, affinities towards the nAChRs for scorpion toxins are in μM range, which makes it unlikely that nAChRs are the main biological targets for scorpion venom.

## 6. Conclusions

Natural products provide a rich source of ligands targeting nAChRs. The venom of snakes and *Cone* snails is especially rich in toxins that have a high affinity (nM range) and selectivity towards nAChRs. For example, α-conotoxins with 3/5 spacing between disulfide bridges, as well as long-chain three-finger α-neurotoxins of snake origin, are potent inhibitors of muscle-type nAChRs. But α-conotoxins with 4/7 spacing that target neuronal types of nAChR often demonstrate similarly high affinities towards multiple kinds of heteromeric receptors. The selectivity of toxins targeting multiple types of receptors could be improved, however. α-conotoxin MII [H9A; L15A], for example, demonstrates selectivity towards α6-containing receptors; ArIB [V11L; V16D] is a selective α7 nAChR antagonist, while PeIA [S9H; V10A; E14N] can discriminate between β2 and β4 receptor subunits in α6-containing receptors. Novel toxins of venomous origin hold great potential for the development of highly potent and selective ligand targeting nAChRs, and they could be extensively used in research.

## Figures and Tables

**Table 2:** Toxins of snake origin targeting nAChRs.

Toxin Same (UniProt)	Snake Species	Target (IC_50_ or Kd Values)	Ref.
α-bungarotoxin (P60615)	Multibanded krait *Bungarus multicinctus* *(Elapidae)*	*Torpedo* nAChRs (IC_50_ = 0.4 nM) hα7 (IC_50_ = 0.4 nM) α7-5HT3 chimera (IC_50_ = 0.95 nM)	[119]
α-cobratoxin (P01391)	Indo-Chinese spitting cobra *Naja naja siamensis* *(Elapidae)*	Ac-AChBP (IC_50_ = 191 nM) Ls-AChBP (IC_50_ = 3.2 nM) α7 (IC_50_ = 0.3 nM) α7-5HT3 chimera (IC_50_ = 9 nM)	[119,125]
αδ-bungarotoxin-1	Blue krait *Bungarus* *candidus* *(Elapidae)*	*T.californica* nAChRs (IC_50_ = 11.4 nM) hα7 (IC_50_ = 1.18 nM) mα1β1δε (IC_50_ = 15.7 nM) mα1β1δγ (IC_50_ = 2.54 nM) (high affinity site α-δ 0.0212 nM on α1β1δε 0.115 nM on α1β1δγ; low affinity sites α-ε = 20.3 nM α-γ = 21.8 nM)	[117]
Drysdalin (F8J2B3)	White-lipped snake *Drysdalia* *coronoides* *(Elapidae)*	rodent α1β1δε (IC_50_ = 16.9 nM) hα7 (IC_50_ = 10 nM) hα9α10 (IC_50_ = 11.3 nM)	[118,126]
Erabutoxin -a (P60775) Erabutoxin-b (Q90VW1)	Chinese sea snake *Laticauda* *semifasciata* *(Elapidae)*	*Torpedo* nAChR (Kd = 0.07 nM) α7 (IC_50_ = 0.5 μ) α7-5HT3 chimera: Erabutoxin a (IC_50_ = 21 nM) Erabutoxin b (IC_50_ = 22 nM)	[120,121,127]
NmmI	Mozambique spitting cobra *Naja mossambica* *(Elapidae)*	α1-γ and α1-δ interfaces (Kd = 100 pM) α1-ε interface (Kd = 100 nM)	[122,123]
κ-Bungarotoxin homodimer (P01398)	Multibanded krait *Bungarus multicinctus* *(Elapidae)*	α3β2 (IC_50_ = 3 nM) α7 and α4β2-weak inhibition	[128,129]
α-cobratoxin homodimer	Monocled cobra *Naja kaouthia* *(Elapidae)*	α1β1δγ of *Torpedo* (IC_50_ = 10 nM) α7 (IC_50_ = 0.2 μM) α3β2 (IC_50_ = 0.15 μM)	[100,101]
Haditoxin (A8N286)	King cobra *Ophiophagus hannah* *(Elapidae)*	α7 (IC_50_ = 0.2 μM) α1β1δγ (IC_50_ = 0.5 μM) α3β2 (IC_50_ = 0.5 μM) α4β2 (IC_50_ = 2.6 μM)	[124]
Fulditoxin	Eastern coral snake *Micrurus fulvius fulvius* *(Elapidae)*	rαβδε (IC_50_ = 2.6 μM) hα4β2 (IC_50_ = 1.8 µM) hα7 (IC_50_ = 7 µM) hα3β2 (IC_50_ = 12.6 µM) reversible blockade	[102]
Irditoxin A and B (A0S864, A0S865)	Brown tree snake *Boiga irregularis* *(Colubridae)*	Species specific activity: avian α1β1δγ (IC_50_ = 10 nM) rodent α1β1δγ (IC_50_ > 10 µM)	[103]
Candoxin (P81783)	Malayan krait *Bungarus candidus* *(Elapidae)*	rα1β1δγ nAChRs (IC_50_ = 10 nM), reversible block rα7 nAChRs (IC_50_ = 50 nM), irreversible block	[97,99]
Weak toxin (WTX) (P82935)	Monocled cobra *Naja kaouthia* *(Elapidae)*	*Torpedo* nAChR (Kd = 90 nM) (native toxin) hα7 (IC_50_ = 14.8 μM) (recombinant) *T.californica* (IC_50_ = 3 μM) (recombinant)	[98,130,131]
Denmotoxin (Q06ZW0)	Mangrove snake *Boiga dendrophila* *(Colubridae)*	Bird specific postsynaptic activity—irreversible inhibition at chick biventer neuromuscular preparation at 10 μg/mL (IC_50_ ~ 300 nM)	[132]
Lc-a (P0C8R7) Lc-b (P0C8R8)	Yellow-lipped sea krait *Laticauda colubrina* *(Elapidae)*	Muscle nAChR LD50 = 0.12 μ/g following intramuscular injection in mice	[133]
Neurotoxin Oh-9 (P83302)	King cobra *Ophiophagus hannah* *(Elapidae)*	IC_50_ on carbachol-induced chicken cervicis muscle contraction—88 nM; Rat adult muscle (IC_50_ = 3.1 μM) Rat fetal muscle (IC_50_ = 5.6 μM)	[134,135]
Waglerin-1 (P24335)	Wagler’s palm viper *Trimeresurus wagleri* *(Viperidae)*	mα1β1δε (IC_50_ = 50 nM, end-plate potential inhibition); mα1β1δγ (IC_50_ = 36 μM); 2000-fold higher affinity to the α-ε (Kd = 9.8 nM) than to the α-δ (Kd = 20.2 μM) binding site interface of the mouse muscle receptor; hα1β1δε (Kd1 = 692 nM, Kd2 = 200 μM) rα1β1δε (Kd1 = 1.1 μM, Kd2 = 36 μM)	[136,137,138]

**Table 3:** The toxins of spider origin, with a suggested and demonstrated activity on nAChRs.

Toxin Name/Spider Species/UniProt	Amino-Acid Sequence	Ref.
HWTX-I *Selenocosmia huwena* P56676	ACKGVFDACTPGKNECCPNRVCSDKHKWCKWKL	[142]
Ω-agatoxin IVA *Agenelopsis aperta* P30288	KKKCIAKDYGRCKWGGTPCCRGRGCCSIMGTNCECPRLIMEGLGLA	[146]
κ-HXTX-Hv1c *Hadronyche versuta* P82228	AICTGADRPCAACCPCCPGTSCKAESNGVSYCRKDEP	[149]

**Table 4:** The amino-acid sequence and potencies of scorpion toxins targeting nAChRs.

Toxin Name/ Species	Structure	IC_50_	Ref.
OSK-1 (α-KTx family)/*Orthochirus scrobiculosus*	GVIINVKCKISRQCLEPCKKAGMRFGKCMNGKCHCTPK	(a) 1.6 μM—mouse muscle; (b) 0.505 ± 0.040 μM—*Torpedo californica*; (c) 20 μM—hα7	[153,154]
HelaTx1 (κ-KTx family)/*Heterometrus laoticus*	SCKKECSGSRRTKKCMOKCNREHGH	(a) 1.4 ± 0.1 μM—*Torpedo californica* (b) 64 ± 7 μM—hα7	[153]
Spinoxin/*Heterometrus spinifer*	IRCSGSRDCYSPCMKQTGCPNAKCINKSCKCYGC	(a) 0.490 ± 0.030 μM—*Torpedo californica*; (b) ≫20 μM—hα7	[153]
